# Supplementary material for: Reconstruction of Ewing Sarcoma Developmental Context from Mass-Scale Transcriptomics Reveals Characteristics of EWSR1-FLI1 Permissibility
Source: Cancers (Basel). 2020 Apr 11;12(4):948. doi: 10.3390/cancers12040948 (PMC7226175; doi:10.3390/cancers12040948)

A) FANCONI ANEMIA PROTEINS

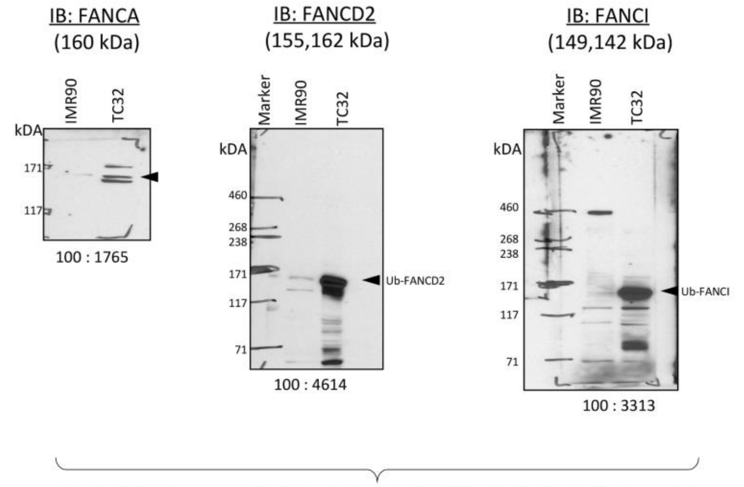

Note: The TC32 band is over exposed in the film (in order to get a band for IMR90) and band density calculation is exaggerated and might not reflect true difference in protein levels

B) FEN1

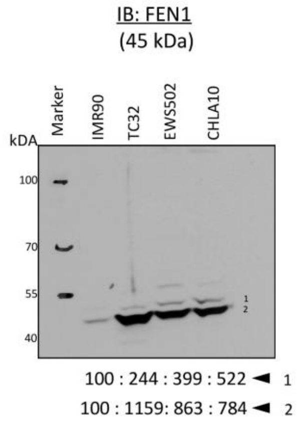

Supplement: Supplementary file 1 [file cancers-12-00948-s001.zip › Supplemental materials/cancers-772370 - WB figures.pdf]
