# Supplementary material for: Reconstruction of Ewing Sarcoma Developmental Context from Mass-Scale Transcriptomics Reveals Characteristics of EWSR1-FLI1 Permissibility
Source: Cancers (Basel). 2020 Apr 11;12(4):948. doi: 10.3390/cancers12040948 (PMC7226175; doi:10.3390/cancers12040948)
Supplement: Supplementary file 1 [file cancers-12-00948-s001.zip › Supplemental materials/Supplemental_Figures.pdf]

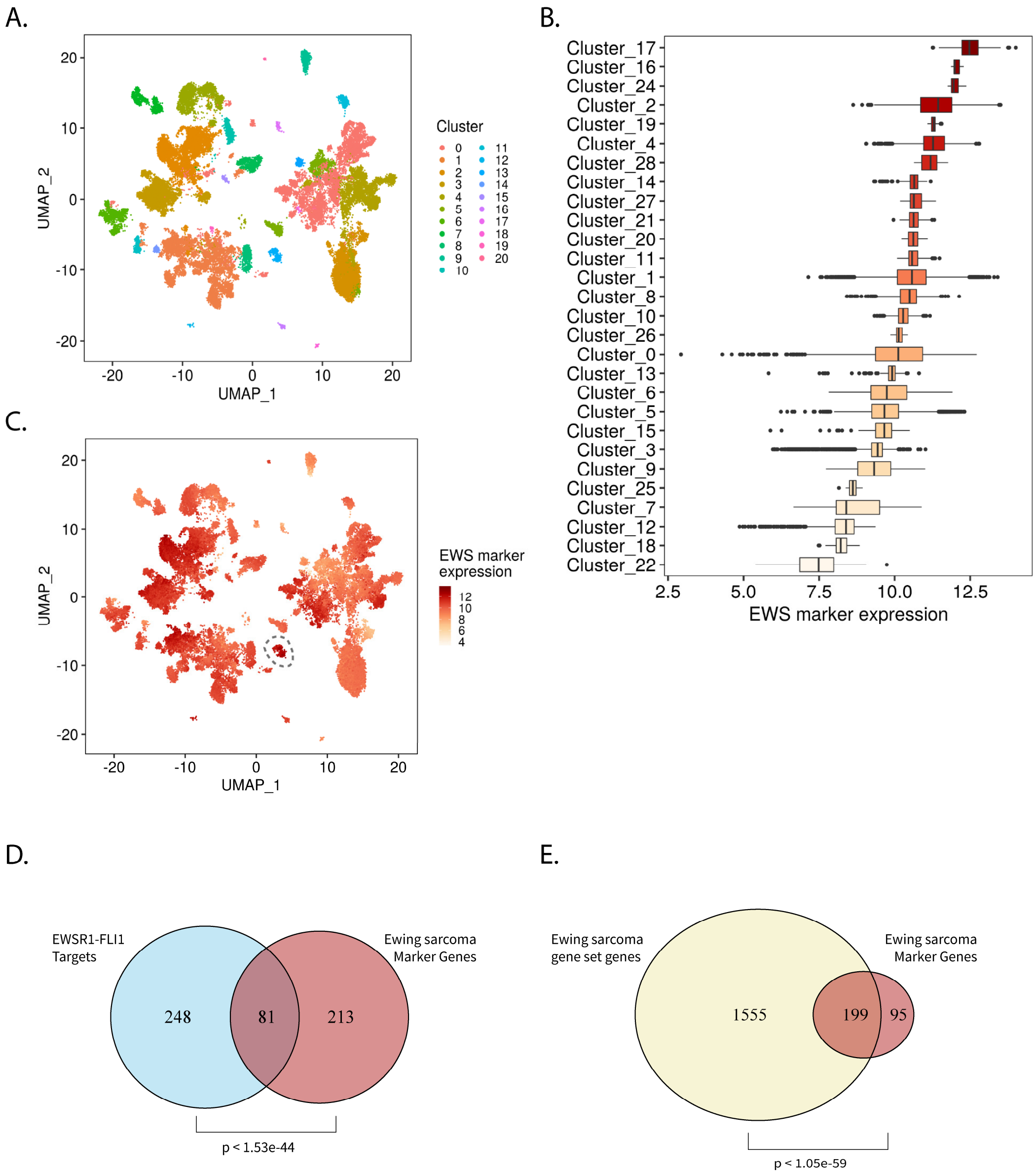

Figure S1. Reconstruction of Ewing sarcoma developmental context from bulk transcriptomics: (**A**) UMAP embedding of bulk transcriptomes with Louvain clusters highlighted; (**B**) Clusters ranked by median Ewing sarcoma marker genes (EWS marker) expression score; (**C**) Overlay of EWS marker expression on UMAP embedding (Ewing sarcoma samples are circled); (**D**) Venn diagram comparing direct EWSR1-FLI1 targets and EWS markers (p value from hypergeometric test); (**E**) Venn diagram comparing Ewing sarcoma gene set genes and EWS marks (p value from hypergeometric test).

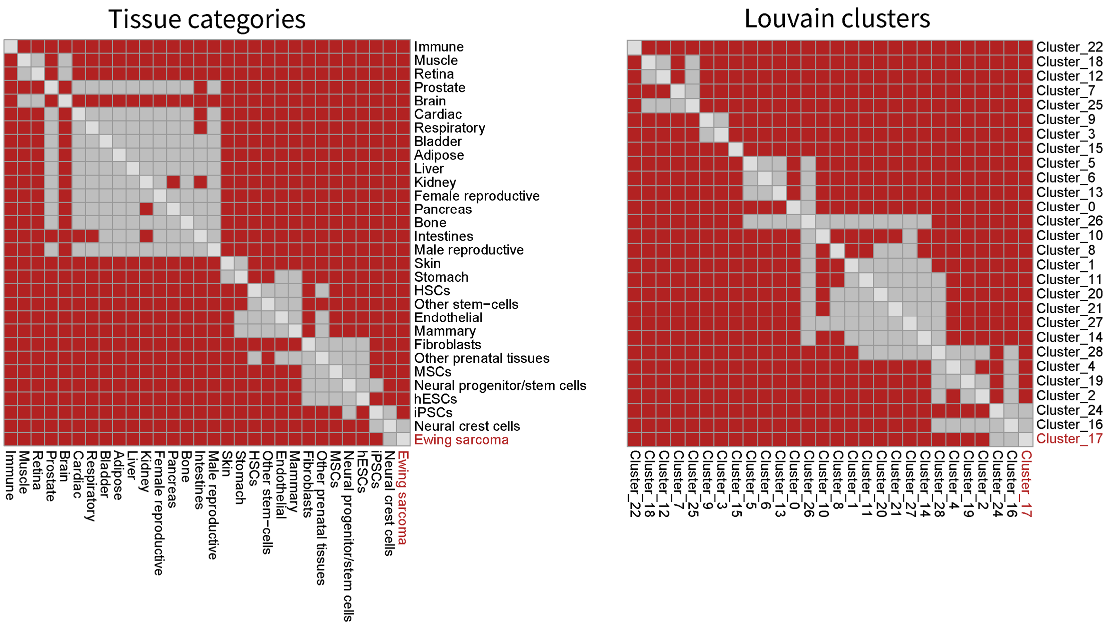

Figure S2. Heatmap showing comparison of Ewing marker expression levels between different tissue types (left) and clusters (right) (two-tailed t test with Holm adjustment; red p <= .05, grey p > .05). Tissues and clusters are ordered by increasing median Ewing marker expression. Red indicates the Ewing marker expression was significantly different between the tissues or clusters in question. Grey indicates that the difference was not significant.

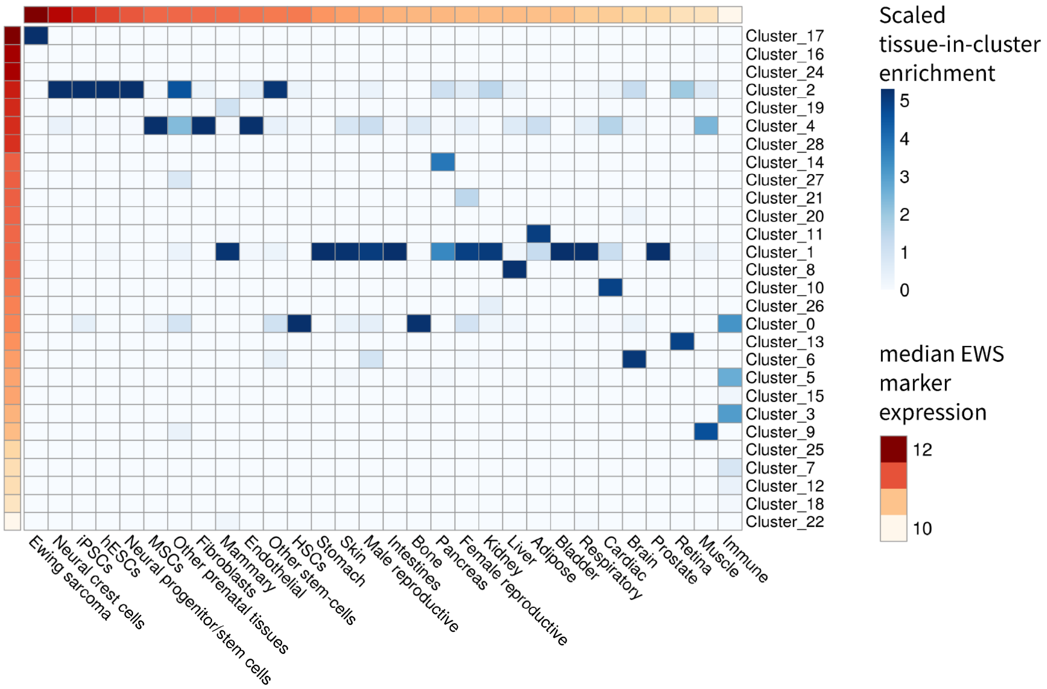

Figure S3. Heatmap showing the scaled enrichment of each tissue type within each cluster. Tissue types and clusters with EWS marker expression (median of VST-transformed and geometric mean normalized read

counts for all EWS marker genes within each sample) sorted by median value within each tissue type and cluster.

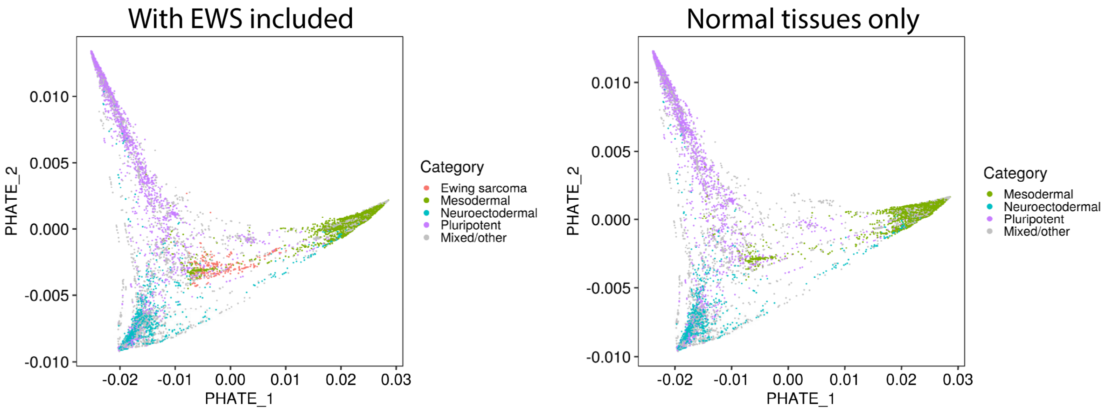

Figure S4. PHATE embedding displaying germ lineage annotations with Ewing sarcoma samples included and recalculated with Ewing sarcoma samples removed.

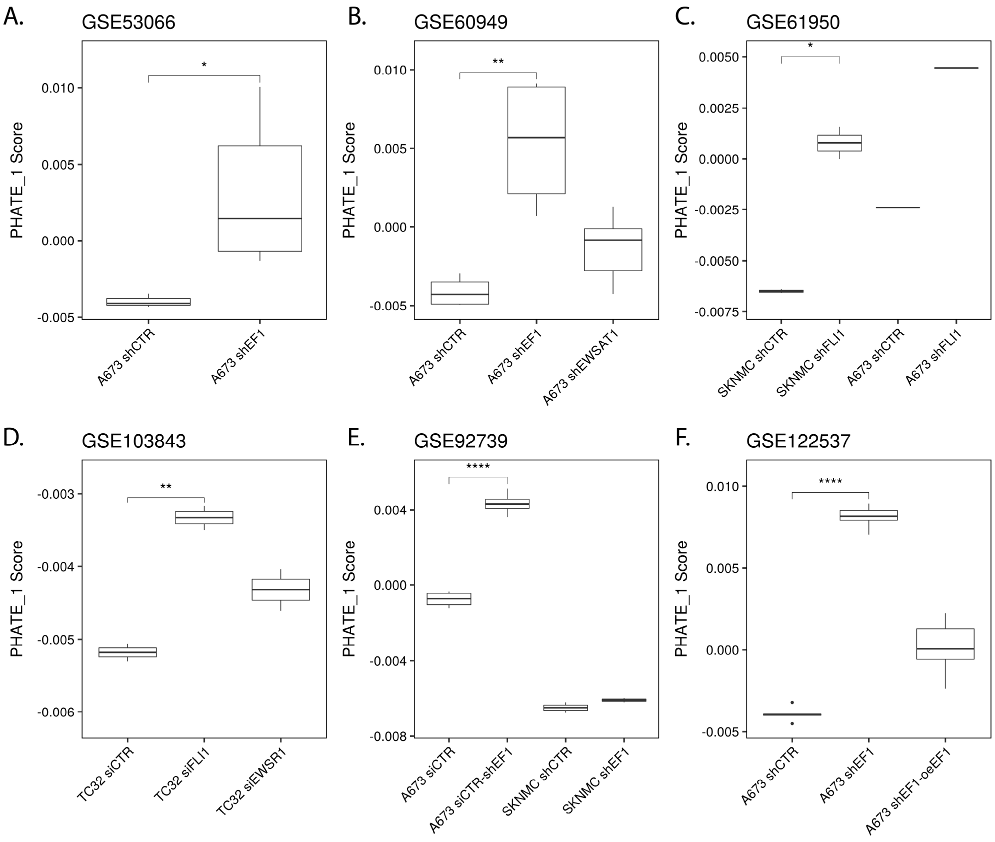

Figure S5. EWS-FLI1 controls the position of Ewing sarcoma within PHATE_1: impact of EWS-FLI1-disrupting interventions on the position of samples within PHATE_1 from (**A**) GSE53066, (**B**) GSE60949, (**C**)

GSE61950, (**D**) GSE103843, (**E**) GSE92739, and (**F**) GSE122537. (one-tailed t test; * p <= 0.05; ** p <= 0.01; *** p <= 0.001; **** p <= 0.0001; ns p > .05)

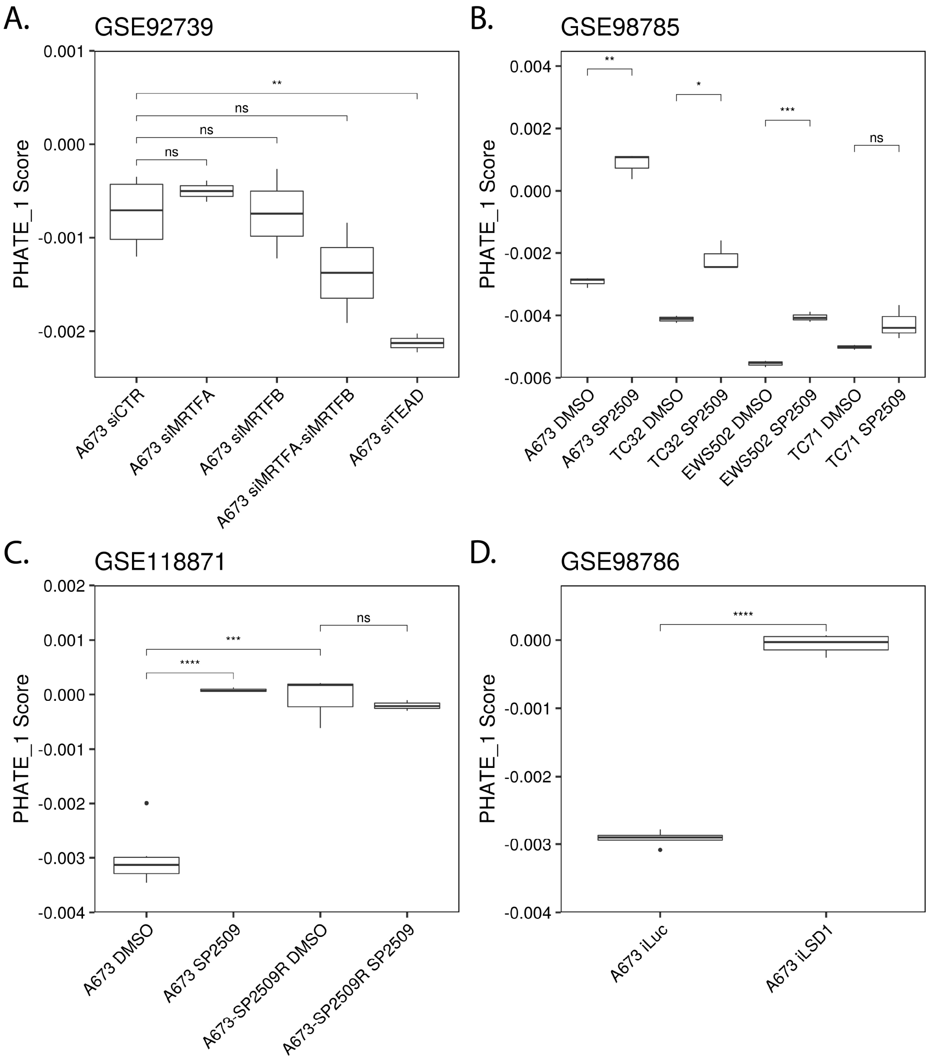

Figure S6. Multiple interventions make a significant impact on position of Ewing sarcoma samples within PHATE_1: Samples analyzed from (**A**) GSE92739, (**B**) GSE98785, (**C**) GSE118871, and (**D**) GSE98786. (two-tailed t test; * p <= 0.05; ** p <= 0.01; *** p <= 0.001; **** p <= 0.0001; ns p > .05)

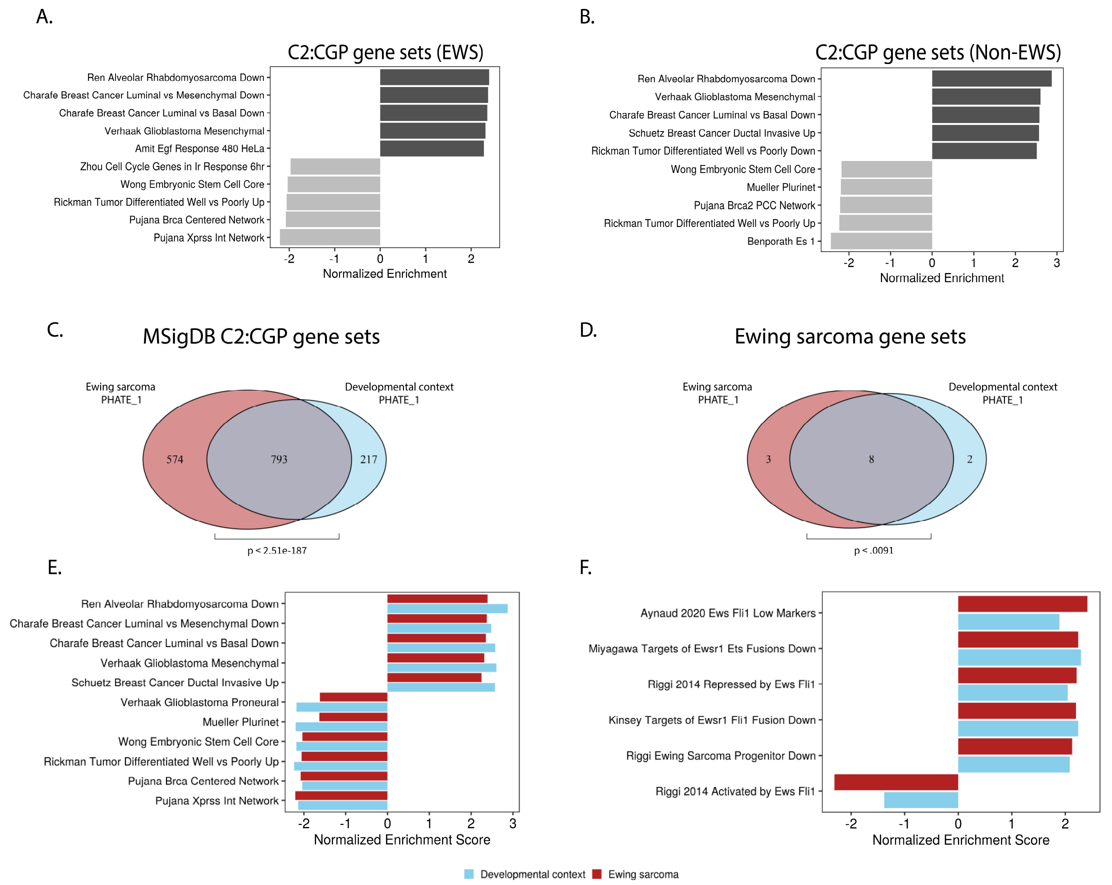

Figure S7. Ewing sarcoma cells traverse underlying developmental trajectories in response to EWS-FLI1 expression and other stimuli: (**A**) Bar-plot showing top C2:CGP GSEA hits with PHATE_1 gene correlations in Ewing sarcoma samples; (**B**) Bar-plot showing top C2:CGP GSEA hits with PHATE_1 gene correlations in developmental context samples; (**C**) Comparison of C2:CGP GSEA hits between Ewing sarcoma and developmental context (p value from hypergeometric test); (**D**) Comparison of Ewing sarcoma-related GSEA hits (p value from hypergeometric test); (**E**) Bar-plot showing top shared C2:CGP GSEA hits between Ewing sarcoma and developmental context; (**F**) Bar-plot showing top shared Ewing sarcoma-related GSEA hits between Ewing sarcoma and developmental context.

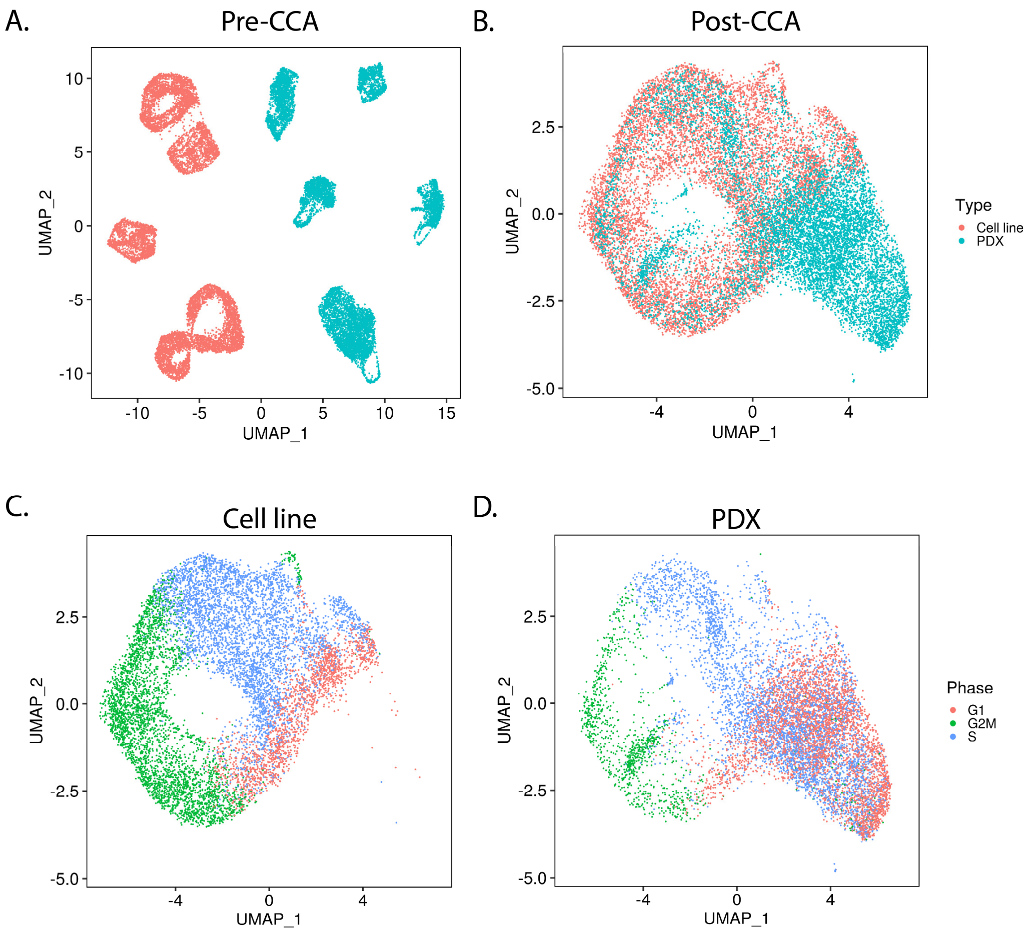

Figure S8. Integrative single cell RNA sequencing analysis of Ewing sarcoma cell lines and PDXs: (**A**) UMAP embedding of Ewing sarcoma cell line and PDX single cells prior to alignment via CCA and (**B**) following alignment; UMAP highlighting cell cycle phase in (**C)** cell line or (**D**) PDX single cells.

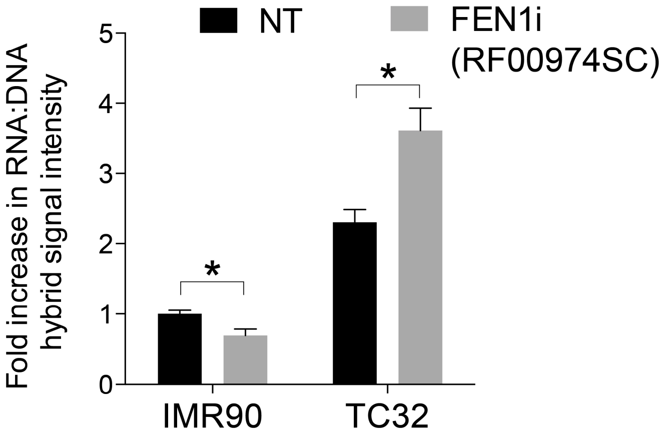

Figure S9. Bar-plot showing effect of FEN1 inhibitor (RF00974SC) treatment on R-loop accumulation in a Ewing sarcoma cell line (TC32) and a fibroblast cell line (IMR90). (* p < .05)
